# Photogrammetric Co-Processing of Thermal Infrared Images and RGB Images

**DOI:** 10.3390/s22041655

**Published:** 2022-02-20

**Authors:** Adam Dlesk, Karel Vach, Karel Pavelka

**Affiliations:** 1 Department of Geomatics, Faculty of Civil Engineering, Czech Technical University in Prague, 16636 Prague, Czech Republic; pavelka@fsv.cvut.cz; 2 EuroGV, spol. s r. o., 11000 Prague, Czech Republic; vach@eurogv.cz

**Keywords:** photogrammetry, thermal infrared image, close-range photogrammetry, thermography, augmented orthophoto, augmented point cloud

## Abstract

In some applications of thermography, spatial orientation of the thermal infrared information can be desirable. By the photogrammetric processing of thermal infrared (TIR) images, it is possible to create 2D and 3D results augmented by thermal infrared information. On the augmented 2D and 3D results, it is possible to locate thermal occurrences in the coordinate system and to determine their scale, length, area or volume. However, photogrammetric processing of TIR images is difficult due to negative factors which are caused by the natural character of TIR images. Among the negative factors are the lower resolution of TIR images compared to RGB images and lack of visible features on the TIR images. To eliminate these negative factors, two methods of photogrammetric co-processing of TIR and RGB images were designed. Both methods require a fixed system of TIR and RGB cameras and for each TIR image a corresponding RGB image must be captured. One of the methods was termed sharpening and the result of this method is mainly an augmented orthophoto, and an augmented texture of the 3D model. The second method was termed reprojection and the result of this method is a point cloud augmented by thermal infrared information. The details of the designed methods, as well as the experiments related to the methods, are presented in this article.

## 1. Introduction

Infrared thermography (IRT) is a non-contact, non-destructive, and non-invasive technique [1] which can be carried out using measuring devices that are able to receive radiation (of a certain wavelength) from an object which has a temperature higher then absolute zero [2]. Thermal infrared cameras are devices which are equipped with a sensor which can receive the thermal infrared radiation of the most external layer of the surface of the object and then create a thermal infrared (TIR) image, also called a thermogram. The TIR image is a matrix where a value of the intensity of the TIR radiation, or the intensity converted to the surface temperature, is stored in each element of the matrix. Then, on the TIR image, it is possible to analyze the temperature of the captured object [3] or the temperature distribution, differences, anomalies, maximum, minimum, etc. The TIR image can also be called as an IRT image [4], but when referring to photogrammetry, the term TIR image is more often used [5,6,7,8]. As is described in published studies, thermography can be used in a wide variety of applications in many different industries.

The main goal of this article is the development of methods aimed to building diagnostics, but in the literature, there are many published applications of thermography. A review of published articles concerned with IRT in the building industry was presented by [3]. In the building industry, relevant applications include surface diagnostics [9], material investigation [10], and energy monitoring [11]. In the building industry, IRT has also been used for object detection on a building [12] and for archaeological purposes [13]. IRT is also widely used in other industries, such as energetics [14], agriculture [15], security [16], zoology [17], seed [18] and plant [19] monitoring, and environmental protection [20]. There is also extensive use of IRT in the medical industry. A review of published articles in this industry was presented by [21].

**Figure 1:**
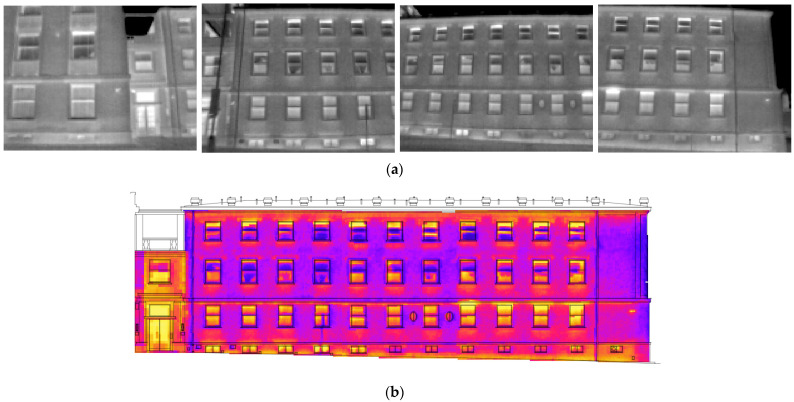
(**a**) TIR images of a building façade. The thermal investigation of the façade on single TIR images is confusing; (**b**) A mosaic created from the single TIR images. The mosaic helps in carrying out improved investigation of the leaks on the façade.

Most of the applications referred to treat TIR images as a simple 2D image [9,13,17,18,19]. This approach is sufficient for many purposes. However, for some applications, further spatial information, such as dimension, orientation, or the area of studied thermal occurrences, may be necessary, e.g., [22]. Adding spatial information to the TIR image can increase the value of the thermal investigation. When the studied object is large (e.g., buildings, urban areas, agriculture fields, glaciers, volcanos), and knowing that TIR cameras usually have low resolution and a narrow field of view, it is necessary to capture the object with tens or hundreds of TIR images. Thermal investigation of the object then becomes enormously complicated or even impossible. When TIR images are processed photogrammetrically, the results of the processing can be an orthophoto, a mosaic (2D) (see Figure 1), a point cloud or a textured 3D model (3D). All the spatial results can be augmented by thermal infrared information derived from the original TIR images. The results are optionally scaled and transformed to the selected coordinate system.

The structure from motion (SfM) method is a prevalent photogrammetric method [23]. Photogrammetric processing of TIR images can also be carried out using the SfM method [24,25,26]. Using these results, it is possible to better understand the studied object, as they enable analysis in three dimensions, automatic or semi-automatic detection of thermal anomalies, the gathering of spatially oriented semantic information, etc.

**Figure 2:**
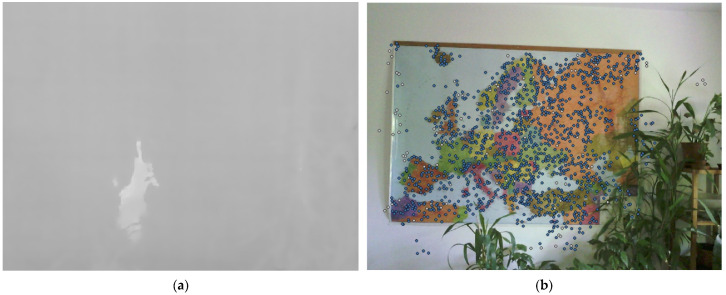
TIR and RGB image of the same scene: (**a**) Feature matching algorithm failed to find any key points or tie points; (**b**) Feature matching algorithm found a sufficient number of tie points (blue dots) [28].

The photogrammetric processing of TIR images involves several challenging difficulties [27]. TIR images have low resolution compared to the common RGB images [7]. TIR images lack visible features or can even be featureless (if there are no temperature changes in the object scene), which significantly complicates, or makes impossible, SfM processing [28]. Algorithms for the SfM method are not able to identify a sufficient number of key or tie points on TIR images, and, as a result, the co-registration of TIR images can fail (see Figure 2).

Another issue is that commonly used photogrammetric targets are not visible on TIR images and, therefore, it is not possible to scale or transform the results. To address the negative factors referred to, several methods of photogrammetric co-processing of TIR and RGB images have been presented in the literature. A review of these methods has been provided in [28]. Generally, for the processing of RGB images, geometric accuracy is used and the results are augmented by corresponding information from TIR images. Overall, the presented methods can be divided into two groups according to their approach. The first approach does not require a fixed system of RGB and TIR cameras and capturing the RGB and TIR images can be carried out completely separately, even at different times. The second approach requires a fixed system of RGB and TIR cameras and, for every captured TIR image, the corresponding RGB image must be captured. The fact that modern hand-held thermal infrared cameras for industrial applications usually have both RGB and TIR sensors, and the camera itself creates a natural fixed system, helps in the development of methods of this approach.

In [24], the authors proposed two methods. Both methods belong to the category in which a fixed system of cameras is required. The first proposed method is called the “sharpening method” and the second method is called the “reprojection method”. The aim of this article is to build on the research published in previous articles [24,28], to describe the sharpening and reprojection methods in detail, and to highlight their specific characteristics. The methods are discussed, and the advantages and disadvantages are pointed out.

A similar approach which is based on the sharpening method was also published by [29]. However, only a plane transformation was used there and, for close-range purposes, plane transformation is not sufficient. Instead of plane transformation, a ray recalculation method has been developed and used. The ray recalculation method is the major improvement for the sharpening method. A similar approach to that of the reprojection method was used also by [6]. The main difference between the approaches is the “test of visibility”, which was designed for the reprojection method. The test of visibility is a key and necessary part of the reprojection method. The test of visibility is described and related experiments are presented in this paper.

### Thermal Infrared Image

This section briefly describes the theoretical background of the process of creating a TIR image. The information on the theoretical background is mainly drawn from [3,30,31,32,33].

Every object which has a temperature above absolute zero (−273.15 °C) emits electromagnetic radiation from its surface. The emitted radiation passes through the ambient atmosphere and can be captured by a device which has a thermal infrared sensor, e.g., a thermal infrared camera. The device captures the radiation in a certain wavelength range and records the intensity of the radiation. Given the parameters of the object and the surrounding ambience, the device can calculate the surface temperature.

The theory of thermography is based on a theoretical object called the black body. The black body is an ideal object which absorbs all incoming radiation and emits all its energy from its surface. According to Planck’s radiation law, the spectral specific radiation $M_{0\lambda }$ of a black body can be described. In Equation (1), c is the speed of light, h is Planck’s constant, k is the Boltzmann constant, T is the temperature in kelvins, $C_{1}=3.74*10^{-16}\;{W\;m}^{2}$ and $C_{2}=1.44*10^{-2}\;K\;m$.

(1) $$M_{0\lambda }=\frac{2\pi hc^{2}}{\lambda^{5}}\frac{1}{e^{\frac{hc}{\lambda kT}}-1}=\frac{C_{1}}{\lambda^{5}}\frac{1}{e^{\frac{C_{2}}{\lambda T}}-1}$$

The spectral specific radiation is dependent on the wavelength for different temperatures. The dependency is presented in [34]. In [34], it is possible to observe that when the temperature of a black body increases, the maximum of the spectral specific radiation is in shorter wavelengths. This phenomenon is described by Wein’s displacement law $\lambda_{max}$, where b=2898 μm*K and T is the temperature in kelvins.

(2) $$\lambda_{max}=\frac{b}{T}$$

By integrating the formula for spectral specific radiation over all wavelengths from 0 to ∞, the intensity of the radiation $M_{0}$ is obtained. According to the Stefan–Boltzmann law, the intensity of the radiation is equal to the fourth power of its absolute temperature. In non-contact measurements, this law is used for the determination of the surface temperature from the thermal radiation.

(3) $$M_{0}=\sigma \;T^{4},\;\;\;\;\;\;\sigma =5.67*10^{-8}\;{\mathrm{Wm}}^{-2}K^{-4}$$

Real objects, unlike a black body, do not emit all their energy. The spectral specific radiation for these objects is given by Equation (4):

(4) $$M_{\lambda }=\varepsilon_{\lambda }M_{0\lambda }$$

where $\varepsilon_{\lambda }$ is the spectral emissivity of the object. The value of the spectral emissivity is between 0 and 1 (the spectral emissivity of a black body is equal to 1). The spectral emissivity is dependent on the wavelength, temperature, material and the angle of the observation. However, there are some objects for which it is possible to consider the emissivity as a constant (in a certain wavelength range). In the literature, these objects are called grey bodies.

If there is radiation towards the object (e.g., the Sun) and the object has a temperature higher than absolute zero, the object:

- absorbs the energy from the source (absorption α),
- reflects the energy from the source (reflectance ρ),
- the energy from the source transmits through the object (transmissivity τ),
- emits the absorbed energy (emissivity ε).

Then ε+ρ+τ=1 and, because most bodies do not transmit in the infrared part of the electromagnetic spectrum, then ε+ρ=1. The emissivity is a key parameter in non-contact temperature measurement. The emissivity of object materials is usually stated in published tables, many publicly available on the internet. When the emissivity is known, the reflectance is given by ρ=1−ε.

In the case of non-contact temperature measurement, a device with a thermal infrared sensor is placed in front of the examined object. The object has a temperature $T_{OBJ}$ and the object is made from material with emissivity $\varepsilon_{OBJ}$. The object emits its own energy through radiant flux $\varepsilon \;{\dot{I}}_{OBJ}$. It goes through the atmosphere with the transmissivity $\tau_{ATM}$, so the radiant flux $\varepsilon \;\tau \;{\dot{I}}_{OBJ}$ goes to the device. All surrounding objects emit the radiation to the examined objects and the examined object reflects the radiation. Afterwards, it goes to the atmosphere and the radiant flux entering the device is $(1-\varepsilon )\;\tau \;{\dot{I}}_{REF}$. The atmosphere also has its own energy and the radiant flux of the atmosphere is $(1-\tau )\;{\dot{I}}_{ATM}$. The summary of these three fluxes entering the device is called the equation of thermography and, using the knowledge referred to above, it is possible to determine the surface temperature of the examined object. Here, it is important to note that determining parameters, such as the emissivity of the object, the reflected temperature, and the transmissivity and temperature of the atmosphere are critically important for surface temperature measurement; insufficient description of these parameters could provide the main source of errors in thermography.

(5) $${\dot{I}}_{TOTAL}=\varepsilon \;\tau \;{\dot{I}}_{OBJ}+(1-\varepsilon )\;\tau \;{\dot{I}}_{REF}+(1-\tau )\;\;{\dot{I}}_{ATM}$$

(6) $${\dot{I}}_{OBJ}=\frac{1}{\varepsilon \;\tau }\;{\dot{I}}_{TOTAL}-\frac{1-\varepsilon }{\varepsilon }\;{\dot{I}}_{REF}-\frac{1-\tau }{\varepsilon \tau }\;{\dot{I}}_{ATM}$$

One of the devices which can capture thermal infrared radiation is a thermal infrared camera. TIR cameras are categorized according to their detector and range of wavelengths. TIR cameras which are used in common industrial applications are usually cameras with a microbolometer sensor [35] as a thermal detector with a wide range of wavelength detection (approx. 2.5 um–20 um). The optics of TIR cameras are made from germanium [36]. The germanium lens steers the radiation to the sensor, which is a grid with resolution much lower than that of sensors of common RGB digital cameras. The resolution of the grid of the TIR cameras is usually from 160 × 120 individual sensors to 640 × 480 individual sensors [28]. Knowing the basic laws of thermography, the software of the TIR cameras recalculates the captured radiation to determine the temperature (possibly in °C).

## 2. Methods

Two methods were proposed in [24,28]. In this section, the methods are described in detail.

### 2.1. Sharpening Method

**Figure 3:**
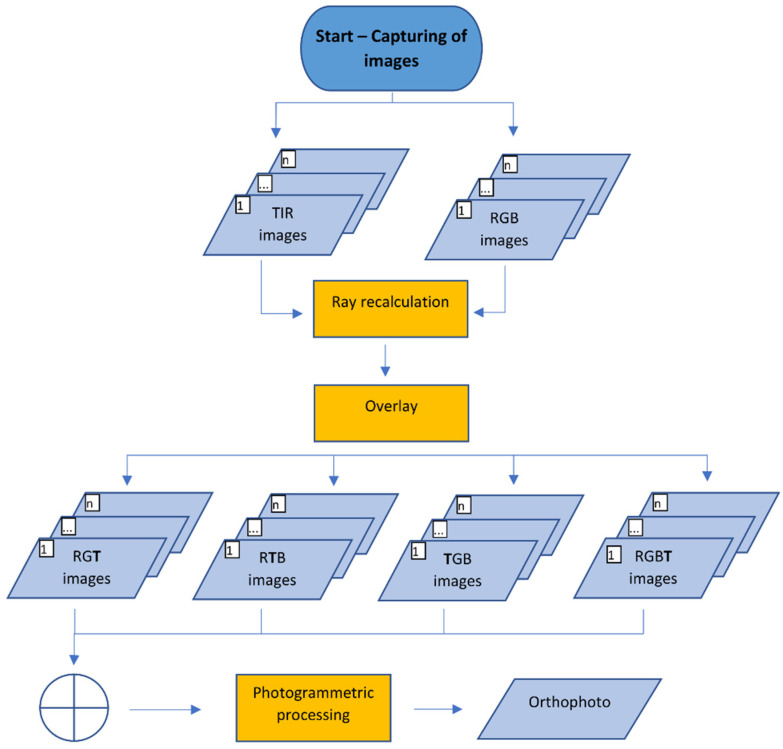
Diagram of the sharpening method.

The sharpening method was designed mainly to create an orthophoto or a texture augmented with thermal infrared information derived from original TIR images. The sharpening method is named after pansharpening but, in the sharpening method, panchromatic images are not used. TIR images are sharpened by the visual features from RGB images captured by common digital cameras. The sharpening method requires a fixed system of TIR and RGB cameras. For capturing the TIR image, it is necessary to capture its corresponding RGB image. In the sharpening method, the images are processed using the SfM method. So, when the images are captured, it is necessary to follow basic capturing instructions for the SfM method. The set of TIR images, and the set of the corresponding RGB images, are the input for the sharpening method. In Figure 3, the workflow of the sharpening method is presented.

At first, thermal infrared values (in °C) in TIR image pixels must be recalculated using a histogram stretching function to 8-bit (0–255) or 16-bit values (0–65,535). The minimum and the maximum of the thermal infrared values should be set the same for all TIR images of the dataset. The minimum and maximum values for the histogram stretching function can be set as actual extremes of the dataset or can be set as the minimum and maximum of the TIR camera thermal range. A large range between the minimum and the maximum can mean that the range of 8-bit values would not be enough (this depends on the TIR camera used). For example, TIR camera FLIR E95 has a temperature range from -20 °C to 120 °C [37]. Recalculating the values to 8-bit values would mean a step of 0.55 °C. This step is not enough for many applications especially when considering that the FLIR E95 camera‘s thermal sensitivity is around 0.04 °C. On the other hand, 16-bit values would mean a step of 0.002 °C. This is less than the thermal sensitivity of the example camera and, thus, there is no need to use a larger range of values than 16-bit.

To overlay the TIR and RGB image and to create a sharpened image, it is necessary to transform the values of the TIR image to the RGB image. A first idea was to transform the information using plane transformation, such as affine or projective transformation. Previously published experiments [28] showed that any plane transformation is not sufficient for close-range purposes. Plane transformation is only sufficient when the images are more-or-less perpendicular to the object, captured over a longer and constant distance from the object, and when the depth of the images is more-or-less constant. However, the authors’ interest is in terrestrial close-range photogrammetry, where there are expected short distances and many changes in the depth of the scene. Because previously published experiments [28] showed that plane transformation is insufficient, a ray recalculation method has been designed.

The ray recalculation method requires parameters of the interior orientation of the TIR and RGB cameras, the relative rotation and translation between the RGB camera and the TIR camera of the fixed system, and depth maps of the RGB images. To obtain depth maps of the RGB images, the RGB images must be preprocessed solely by any photogrammetric method, possibly the SfM method. When the 3D triangulated model or the point cloud is generated and scaled, the depth maps are rendered onto one of the generated results.

Having all the required inputs, the first RGB image of the dataset is taken. Then a blank raster with the same dimensions as the RGB image taken is created. The goal is to remap the corresponding TIR image to the blank raster image and to fill the blank pixels with the thermal infrared information from the TIR image. The process of the remapping is described below.

**Figure 4:**
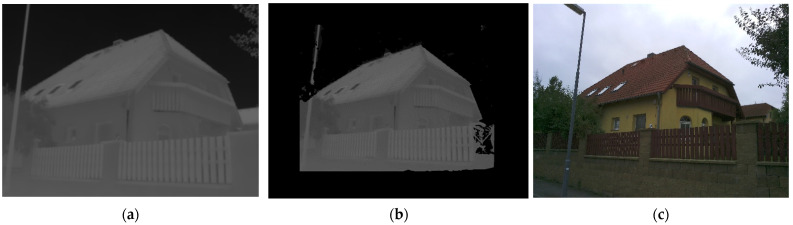
(**a**) Original TIR image; (**b**) TIR image remapped by a ray recalculation function with the same resolution as RGB image; (**c**) Original RGB image; the remapped TIR image and RGB image can be overlayed and merged.

For the first pixel of the RGB image, a ray from the optical center of the RGB image through the undistorted position of the pixel is computed. A point which lies on the scene, and which is projected to the pixel of the RGB image, must be found on the ray. The ray itself is infinite, but knowing the depth information derived from the corresponding pixel of the depth map, the 3D coordinates of the point on the scene in the RGB image camera coordinate system is possible to compute using the basic similarity of triangles. The coordinates of the point are possible to transform to the TIR camera coordinate system (the rotation and translation between the RGB camera and the TIR camera for a fixed system is known). The point is then reprojected from the TIR camera coordinate system to the TIR image (the internal orientation of the TIR camera is also known). The pixel value of the thermal infrared (the TIR image is already recalculated to 8-bit or 16-bit values) is stored to the pixel of the blank raster (the same position as the examined pixel of the RGB image). The process is then repeated for every pixel of the RGB image and the remapped TIR image is created (see Figure 4).

**Figure 5:**
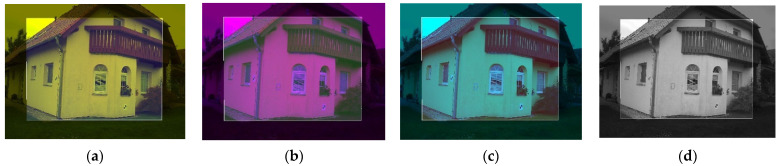
Sharpened images: (**a**) RGT combination; (**b**) RTB combination; (**c**) RGT combination; (**d**) RGBT combination.

Then, the remapped TIR image with 8-bit values can replace one of the channels of the RGB image (with 8-bit channels) and create the sharpened image. For example, from the RGB image, the blue channel is replaced by the remapped TIR image which creates the RGT image (see Figure 5a), where visible features of the scene (channels, R and G) and thermal infrared information (in channel T) are partly stored. This could be applied analogously and the RTB or TGB sharpened image can be created (see Figure 5b,c). The sharpened image should be stored in an image format without compression so as not to change the data in the T channel. It is possible to process these 3-channel images in any photogrammetric software because the images have the same structure as common RGB images. In advanced photogrammetric software e.g., Agisoft Metashape [38], there is support of multi-channel images with, e.g., 16-bit values of a band. Then, it is possible to use the remapped TIR image with 16-bit values and to add the channel to the RGB image, which must also be recalculated to 16-bit. Then the RGBT (Figure 5d) image, with full visible features (channels RGB) and thermal infrared information (channel T), is created. Every image of the dataset should be sharpened under the same scheme (e.g., RGT). The fact that the visible information from RGB images is present in all combinations (at least partly) helps the algorithms to better find the key and tie points and eliminates the problem with photogrammetric targeting. In this case, standard photogrammetric targets can be used and are easily identifiable in the images. The question of which combination is the most sufficient to use for SfM processing has been raised and, to answer the question, an experiment was performed (see Section 3.1).

When the dataset of sharpened images is ready, the process continues with standard photogrammetric processing with the SfM method. The results of the processing can be a colored point cloud, a textured 3D model or an orthophoto. The color of the point cloud is usually weighted in some way, meaning that we would not have the original values from the TIR image. When creating texture for the 3D model or the orthophoto, usually the software has an option to prevent averaging or weighting of the colors. Because of this, the sharpening method is more suitable for an augmented orthophoto or textured 3D model. In the case of an orthophoto and texture of the 3D model, it is possible to recalculate the values of the T channel back to the original thermal infrared information. By adding the original thermal infrared information to the pixels, the spatial results augmented by the thermal infrared information are created. For a point cloud, augmented by the thermal infrared information, it is more convenient to use the reprojection method.

### 2.2. Reprojection Method

**Figure 6:**
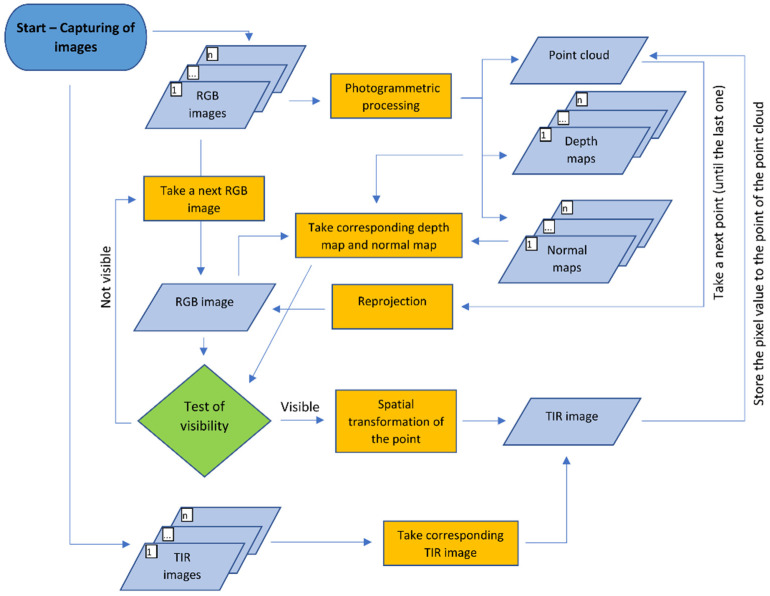
Diagram of the reprojection method.

The reprojection method was designed primarily to create a point cloud augmented with thermal infrared information which is derived directly from a TIR image. The sets of TIR images and corresponding RGB images captured by the fixed camera system are the inputs of the reprojection method. The images should be captured according to the common suggested capturing scenarios for SfM. Relative rotation and translation between the RGB and TIR camera coordinate systems, and the parameters of the interior orientation of both cameras gathered by geometric camera calibration, must be known. In Figure 6, the workflow of the reprojection method is shown.

The first step of the reprojection method is SfM processing of the RGB images only. This is an essential advantage of this method, because the TIR images do not enter SfM processing at all; thus, all the negative factors referred to above are naturally eliminated. After SfM processing, the resulting photogrammetric model is scaled or transformed by control points. After scaling, the point cloud is generated. For the subsequent steps of the reprojection method, it is necessary to generate the depth map and normal map of each processed image. The depth maps and normal maps can be rendered on the point cloud, or on the 3D triangulated model generated based on the point cloud.

Then every point of the point cloud should be augmented by thermal infrared information. The algorithm takes a first point of the point cloud and reprojects the point to the camera coordinate system of the first taken RGB image from the set of RGB images which were used in SfM processing (the external orientation parameters of the images are known). Knowing the parameters of the interior orientation of the RGB camera, the point is transformed from the camera coordinate system to the RGB image. Even though the point is possible to reproject to the RGB image, this does not mean that the point belongs to the scene in the image (the point can be physically behind the image scene). To check if the point is visible in the image, it is necessary to carry out the test of visibility. The test of visibility requires information about the scene. The information about the scene is given by the depth map and the normal map of the image. The correct test of visibility is the key part of the reprojection method.

For the test of visibility in the reprojection method, four tasks have been designed. The first task is to check if the projection of the point of the point cloud is within the RGB image dimension. Knowing the exterior and interior orientation of the RGB image, the point is simply reprojected to the RGB image and the coordinates in the images are read and compared. If the reprojected point is within the image dimension, then the depth test is carried out. For the depth test, it is necessary to create a depth map of the RGB image. The position of the reprojected point of the point cloud to the RGB image is used also for the depth map image and the depth value from the corresponding pixel is read. Then the test compares the Z-coordinate of the point transformed to the RGB camera coordinate system to the depth value from the pixel. If the difference is within a certain tolerance, the point is still considered as potentially visible. The question is how to set the tolerance. The depth test is easy to implement, and it is very efficient for points which are not clearly visible; however, the reliability of the test decreases when the distance differences are closer to the tolerance. In close-range applications, the tolerance will have an important effect when deciding the visibility of the point (especially parts behind the corners and on edges of structures, such as windows and door jambs). Because of this uncertainty, a normal test as a third task of the test of visibility should be added. The third task of the test of visibility is the normal test. For the normal test, the normal maps of RGB images must be rendered. In each pixel of the normal map the normal vector converted to RGB values is stored. For the normal test, it is necessary to also have a normal vectorof each point of the point cloud. In common photogrammetric software, it is possible to store the normal vector of each point to the point cloud. The laser scanning results also provide a normal vector of each point. When the normal vector is not present in the point cloud, it is possible to calculate it in other software, such as Cloud Compare [39]. The normal vector of the point taken of the point cloud must be transformed to the camera coordinate system of the RGB image. If the point of the point cloud is reprojected to the RGB image within the image dimension, then the image coordinates are used, and the pixel value of the normal map is read. The normal vector of the point is then compared to the values read in the normal map. The vectors are compared according to cosine similarity. If the angle between the two 3-dimensional normal vectors is below a certain tolerance, the point of the point cloud is still considered as visible, and the visibility test continues with the last task. Theoretically, the normal test can be removed, but it requires a very accurate point cloud and very accurate parameters of exterior orientation of the RGB images. As a fourth task, the point of the point cloud is transformed from the camera coordinate system of the RGB image to the camera coordinate system of the TIR image and the point is reprojected to the TIR image. If the reprojected point is within dimensions of the TIR image, the point is considered as visible, and the process of the reprojection method continues for other points of the point cloud, or for other images.

If the test of visibility proves that the point is truly visible on the RGB image, then, knowing the relative rotation and translation between the RGB and TIR cameras, the point is transformed from the RGB camera coordinate system to the TIR camera coordinate system of the corresponding TIR image. From the TIR camera coordinate system, the point is projected to the TIR image. The value from the pixel where the point was projected is read and stored to the point of the point cloud. Then the point is reprojected to the second RGB image of the RGB images set and so on. If the point is visible in more than one image, more thermal infrared values to the point of the point cloud are stored. The final thermal infrared value is then calculated as a mean of the set of values; there is also the possibility to statistically detect outliers and remove them from the set of values.

The advantage of this method is that, in the process, it is possible to replace the point cloud generated from RGB images with a laser scanning point cloud referenced in the same coordinate system. A point cloud from a laser scanner can have higher accuracy or density compared to the point cloud generated from RGB images. Then, the process remains unchanged and the result of the reprojection method is augmented by a laser scanning point cloud. When a laser scanning point cloud is used for the reprojection method, the depth and normal maps should be rendered on a 3D triangulated model, because the model is continuous. The main disadvantage of the reprojection method is that the computation of the method (when there are a lot of points of the point cloud and many images) takes many hours. To linearly reduce the processing time, it is not necessary to use all the RGB images in the set, but it is enough to use only selected images. For example, to process the point cloud of a building façade, it is not necessary to use all 30 images from which the point cloud is created, but it is sufficient to manually select just 3 or 4 images, if it is certain that each point is visible in at least one image. However, this requires manual selection input which precludes automatization of the processing.

## 3. Experiments

In Section 2, several questions were raised regarding the presented methodologies. To answer these questions, experiments were carried out and are presented in this chapter. For the experiments, TIR camera FLIR E95 was chosen. FLIR E95 is a camera equipped with a thermal infrared sensor and an RGB sensor and a fixed camera system is created which is required for the processing using sharpening and reprojection methods. The general parameters of FLIR E95 cameras are detailed in Table 1. Both cameras of FLIR E95 were geometrically calibrated using a three-level spatial calibration field. The relative translation and rotation between the RGB and TIR camera coordinate systems was also computed on the three-level spatial calibration field. The result of geometrical calibration and the relative translation and rotation is presented in Table 2 and Table 3. The process of gathering the resulting parameters, as well as the impact of the accuracy of the resulting parameters to the transformation processes of the suggested methods, is presented in [28]. The TIR camera was radiometrically calibrated by the manufacturer.

### 3.1. Sharpening Method–Test of Image Band Combinations

The question of which combination of image bands is the most suitable for SfM processing has been raised. An experiment was carried out. Three image samples were chosen, with each image sample capturing a different part of the object which was a family house. Initially, the RGB images of three samples were processed solely in Agisoft Metashape. The RGB images were masked to have exactly the same computational area as the masked images of different combinations (i.e., RGT, RTB, TGB, RGBT). The images were co-registered using their full resolution. Then the point cloud was generated. The number of tie points (TP) and the number of points in the point cloud (PC) were noted. The process was repeated in the same way for all four combination of image bands (also masked), and the numbers of TP and PC were compared as percentages (see Table 4) to the numbers resulting from the RGB image processing. As a last step, the TIR images were also converted to .bmp image format and processed, and the numbers of results were also compared. In Agisoft Metashape, it is possible to set a key point or tie point limit for co-registration. In our case, no parameter limit was set, so, in Table 4 the maximum possible tie points are provided.

According to Table 4, it is possible to see that the image band combination RGT gave the best results. Most of the numbers are almost the same as the results given by processing the RGB images. The remaining combinations gave numbers around 80% of the numbers for RGB image processing. The RGBT combination compared to the RGT combination gave the worse results, but the advantage of a lesser temperature step and full colour information still prevailed there. The processing of TIR images showed a very low number of tie points and points of the point cloud. It must be noted that the resolution of TIR images is lower than the resolution of RGB images so that the comparison is not fair. The numbers are still presented to illustrate that some co-processing with RGB images is necessary. Even though all TIR images were co-registered, the number of tie points was very low. The point cloud generated based on TIR images was too thin and insufficient even on first impression.

### 3.2. Test of the Visibility–Experiment

The aim of this experiment was to determine what is the best way to set the tolerances of the test of the visibility. For this experiment, part of the building façade was chosen as a test scene. The scene was captured by thermal infrared camera FLIR E95 and for each corresponding RGB image, the TIR image was captured. The methods of this article are focused on close-range photogrammetry, so the façade was captured at a distance of 2–4 m. The average sample distance of the images was approximately 1.3 mm/pix. Overall, 24 images were captured. On the façade, four photogrammetric targets were placed and used as control points. The targets were measured by a total station just from one station. The images were processed using the SfM method with the commercial photogrammetric software Agisoft Metashape. For image co-registration, full image resolution was used. The average tie point residuum in the images was 0.93 pix. The photogrammetric model was transformed to the targets and the RMSE_xyz_ on control points was 0.001 m (approx. 1 pixel). After georeferencing, the point cloud and 3D textured model were generated. Again, the full resolution of the images was used for point cloud generation. The experiment was performed on the points of the point cloud. The depth maps and normal maps have been rendered on a 3D model.

**Figure 7:**
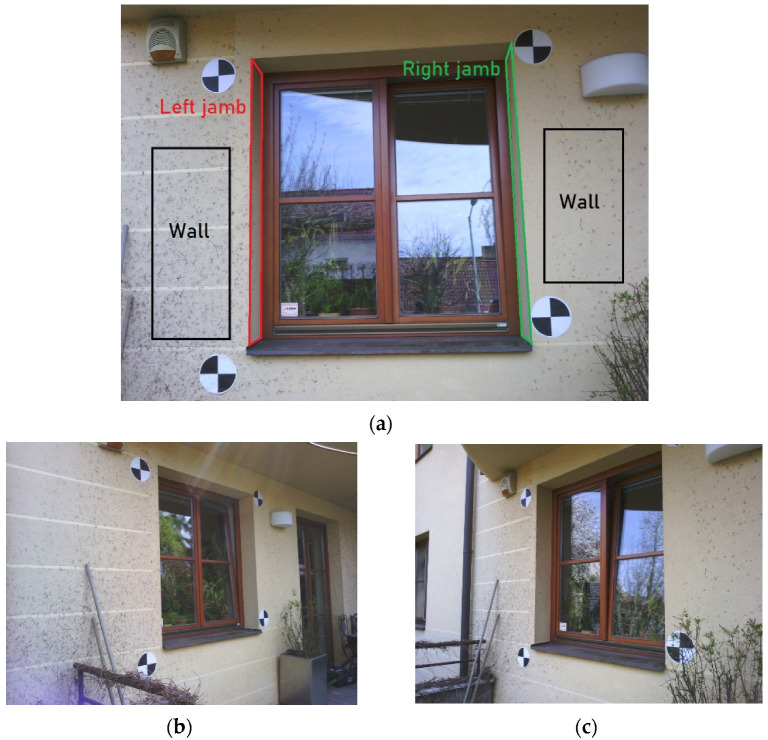
(**a**) Centre image. In the figure, the positions of the points of the three groups of point cloud (wall, left jamb, right jamb) are shown; (**b**) Left image; (**c**) Right image.

**Figure 8:**
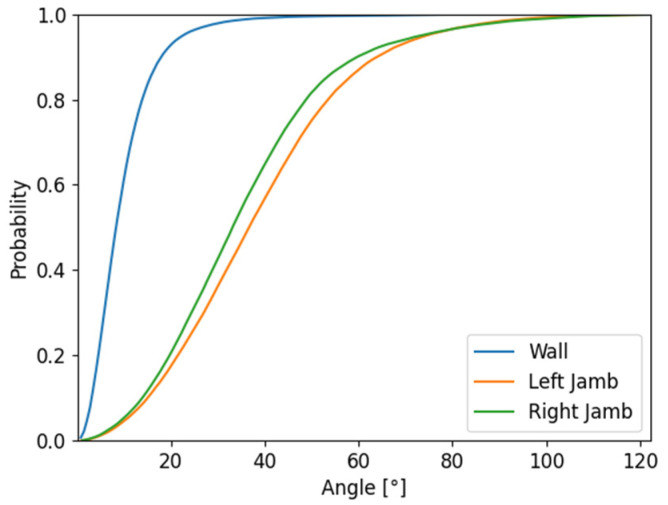
Cumulative distribution graph of set of angle differences.

The tolerances in the depth test could have been set according to the accuracy indicators of the photogrammetric model, e.g., sample distance, RMSE values of the control or check points. In this experiment, the tolerances were set according to the RMSE_xyz_ on the control points. The RMSE_xyz_ on the control points was 0.001 m. The tolerances have been set as RMSE_xyz_ multiplied by 5, 10 and 15. The tolerances for the experiment were 0.005 m, 0.010 m, 0.015 m. It is more difficult to decide the angle tolerance in a normal test. The normal vectors usually have higher variance, especially at surfaces of the model, which are either textureless or not ideally covered with sufficient image overlap or by the images. It is possible to set the tolerance by a rough estimate or by deeper examination of the variance of the normal vectors of the point cloud. To examine the variance of the normal vectors on the point cloud, three groups of the point cloud were manually created. Only points which represented a plain façade wall (W) belonged to the first group. Only points which represented left window jamb (LB) and right window jamb (RB) (see Figure 7a) belonged to the other two groups. All three parts were considered as planar and similar normal vectors were expected at all points of each group. In each group of points, an average normal vector, as a mean of the x, y, z components of the normal vector of all points of the group, was computed. Then, every normal vector of every point of the group was compared to the average normal vector by cosine similarity and the angle between the vectors was calculated. The distribution of the set of computed angles is presented in a cumulative distribution graph in Figure 8.

According to Figure 8, the normal vector of the points varied. On the part of the point cloud which represents the wall, the variance of the normal vector was the lowest and the angle tolerance for the normal test could be set between 20°–30° to cover most of the points. A more problematic situation was on the jambs. These parts did not have ideal intersection angles in the images, so the variance of the vectors was higher. To cover around 80% of the points, the angle tolerance should be set between 50°–70°. This wide-angle tolerance can lead to wrong indications if the point is visible or not. For practical application, it is more important to ensure correct indication of the visibility even when a certain percentage of points is lost, rather than to indicate the point as visible when it is not. That would lead to incorrect thermal augmentation of the points and the final result would be misleading.

For the following experiment, three images of the dataset were chosen. The left image captures the window from the left side, the center image is perpendicular to the façade and the right image captures the scene from the right side. For each image, points of the groups were reprojected to the images and the visibility test with various tolerances was carried out. The results of the experiment of the visibility test are presented in Table 5. In the table, when the group of points is visible in the image is noted. Then, the depth tests and normal tests were carried out with various tolerances. According to the RMSE_xyz_ on the control points on the photogrammetric model, 5 mm, 10 mm and 15 mm tolerances were set for the depth test. According to the examination of the variances of the normal vectors of the point cloud, 25°, 40° and 60° tolerances for the normal test were set.

From Table 5, it is possible to observe high confidence when the wall group of points was reprojected. Both tests for all images show that they were very successful even using the lowest tolerances.

The depth test showed the worst results when testing the wall group of points reprojected in the right image with the lowest tolerance (72%). The image captures the group of points at a very acute angle. The capturing angle has an effect on the results of the test of visibility, especially for the depth test. This effect is possible to observe at the left jamb and right jamb points in the center image. Even though all the points of the left jamb and right jamb group are visible, the depth test was able to recognize the visibility only in 49% of points on the left jamb when the largest 15 mm tolerance was set. The right jamb is captured at an even more acute angle, so the depth test recognized the visibility only in 21% of points. When the points of the left jamb were tested in the right image (the right image axis is approximately perpendicular to the plane of the left jamb), the depth test was successful in 97% of points, and when the points of the right jamb were tested in the left image (also perpendicular angle), the depth test was successful in 99% (using the 15 mm tolerance) of points. The testing showed that when the image captures the group of points with normal vector towards the camera, the depth test is confident even with the middle tolerance of 10 mm.

The normal test looks independently at the angle of capture of the object. According to the results, the normal test is dependent of the variance of the normal vector in a group of points. The results correspond to the cumulative distribution function in Figure 8. For example, the normal test on points of the left jamb with a tolerance of 40° showed 48% in the center image and 72% for the right image, respectively. According to the cumulative distribution function, the expected value was 56%. Because of the large variance of the normal vector on jambs, the normal test in some cases falsely determined points not visible as visible. With the 60° tolerance, the normal test wrongly determined 27% of the points on the left jamb in the left image and 25% of the points on the right jamb in the right image. Fortunately, in these cases, the depth test is reliable in detecting not visible points, and, when both tests were applied together, there was only 1% of points wrongly determined as visible, and 0% on the right image, respectively.

To conclude, it is not straightforward to set the proper tolerances for the depth and normal tests. It is not possible to give general advice. The tolerances depend on each project, on its purpose and the demands placed. It is not possible to set a general rule. For some of the projects it is possible to set low tolerances for the test. In this case, a lot of points of the point cloud could be lost. However, in some of the applications, where it is important to obtain accurate temperature data, it is a better solution to lose a certain amount of points rather than to have the full recalculated point cloud with potentially wrong temperature values. It is advisable to set lower tolerances when there is a larger dataset of testing images.

### 3.3. The Sharpening and Reprojection Method Used on the Test Object

**Figure 9:**
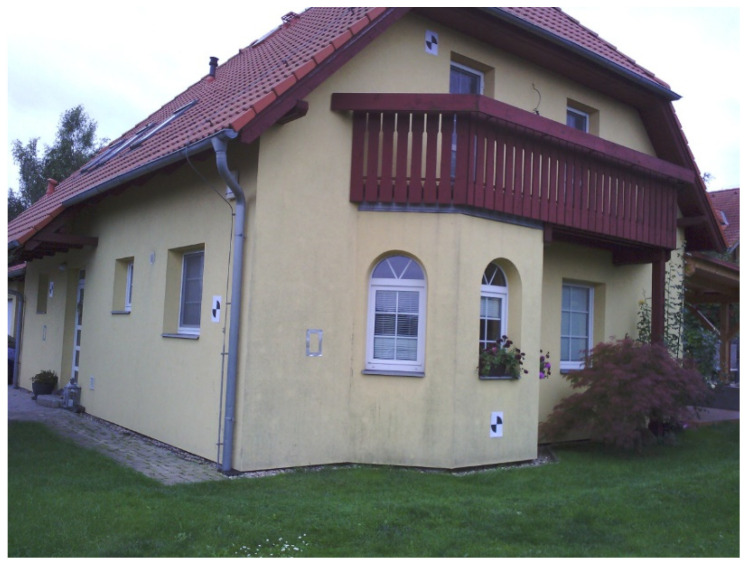
The test object.

To test the proposed sharpening and reprojection methods, an experiment was carried out. As a testing object, a family house was chosen (see Figure 9). The family house is inhabited, so parts of the building are covered by vegetation and other regular objects. The family house facade is isolated, so there were not expected to be temperature changes. The family house is colored in one color and the facades are textureless. Due to these two facts, there was presumed to be a difficulty with processing of images using the SfM method. However, the object was chosen because it represents a common real object. On the object black and white targets were placed for scaling and orientation of the results. The targets were measured by a total station.

#### 3.3.1. Capturing the Images

The process of capturing the images was carried out according to the suggestion of the local distributor of the FLIR E95 camera. At first, a day with cloudy weather and stable air conditions was chosen. As input to the camera, multiple parameters were measured and set in the TIR camera. The air temperature and humidity were measured using a calibrated thermometer and hygrometer. The reflected ambient temperature is a very important parameter. Crumpled aluminum foil was placed on the object and the average temperature of the foil was measured by the FLIR E95 camera and used as the reflected ambient temperature. The reflected ambient temperature was determined at 3.1 °C and the main source of the reflectance was the cloudy sky. Because the weather was constant during capturing of the images, the reflected ambient temperature was expected also to be constant. This was confirmed by measurement at the end of image capturing when the reflected ambient temperature was measured at 3.0 °C. After parameter setting, the object was captured by the FLIR E95 camera in different positions and angles to cover every part of the object in multiple images. For every TIR image, a corresponding RGB image was captured. In total, 187 image pairs of corresponding images were captured. The average sample resolution of the RGB image was 3.2 mm/pix. On the object, five surface temperature check points were established and the surface temperature of the façade was measured using a calibrated touch thermometer. The temperatures were compared to the temperatures in the captured TIR images. The values did not exceed 0.9 °C which is below the accuracy of reading the temperature of the FLIR E95 camera signifying that all parameters were set correctly.

#### 3.3.2. Processing Using the Sharpening Method

The sharpening method was used to create TIR orthophotos of the facades of the objects with the temperature value stored in each pixel of the orthophotos.

Initially, only RGB images were processed in the Agisoft Metashape software. For the relative orientation computing, the full resolution of the images was used. The average residuum of tie points in the images was 1.3 pix. To scale the model, the relative orientation was referenced using 10 control points. The RMSE_xyz_ of the referencing was 6.5 mm. After referencing, the point cloud was generated. It was obvious that some parts of the building with very low texture were not ideally described by the point cloud. To partially improve the result, lower filtering was used when the point cloud was generated. From the generated point cloud, the 3D triangulated model was generated. The model was deformed in only some of the textureless parts. On the model, the scaled depth maps of the images were generated. It was necessary to prepare the following data:

- The set of RGB and TIR images;
- Parameters of the interior orientation of the RGB camera and the TIR camera;
- Relative translation and rotation between the RGB camera and the TIR camera;
- Depth maps for the RGB images.

**Figure 10:**
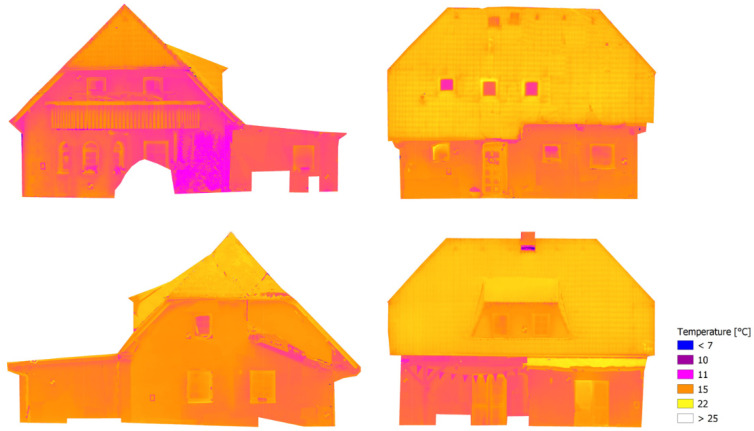
The resulting orthophotos augmented by thermal information.

The group of TIR images, the corresponding RGB images and the corresponding depth maps were prepared for the process of ray recalculation. Based on the created depth maps and the known parameters of the interior orientation of both cameras and the known relative translation and rotation between the cameras, 4-channel RGBT images were created. In the fourth channel, temperature information recalculated using the histogram stretching function to 65,536 values (16-bit) was stored. The recalculated images were processed by Agisoft Metashape. First, the relative orientation was computed. In some pixels of the RGBT image there was a “no data” value in the fourth channel. Those parts were covered by a mask and then there were no calculated tie and key points. The model of the relative orientation was referenced using five control points (equally distributed on the object). Five check points were used for the accuracy check. The average tie point residuum in the images was 1.9 pix. The RMSE_xyz_ of the referencing was 0.6 pixels, which signifies relatively good accuracy of the relative orientation. However, the RMSE_xyz_ on check points was 17 mm. This error was probably caused because measurement of the coordinates of the targets by total station was carried out from multiple stations. After the accuracy check, the 3D textured model was created and, using the model, four orthophotos (one for each façade) were generated. To achieve unchanged temperature values, every averaging of the pixel values (colors) was disabled during orthophoto generation. The fourth channel of the RGBT orthophoto was separated and recalculated back to the temperature values, using the inverse histogram stretching function, and TIR orthophotos were created (see Figure 10). The TIR orthophotos were visualized in QGIS [40]. Some parts of the façade, which were covered by some other object, were cropped on the orthophotos.

#### 3.3.3. Processing Using Reprojection Method

The reprojection method was used to create a point cloud of the object where a temperature value is stored in points of the point cloud. The reprojection method can use a point cloud generated by photogrammetric processing or a point cloud acquired by laser scanner. For this experiment we chose a point cloud acquired by laser scanner.

The testing object was laser scanned using a Leica BLK360 laser scanner. The point cloud from the laser scanner was processed in a Cyclone Register 360 and the point cloud was referenced to the seven control points. The RMSE_xyz_ of the referencing on the control points was 0.012 m. It was then necessary to process the RGB images to obtain their absolute exterior orientation. The model from RGB images was already computed for the sharpening method and the details are presented in Chapter 3.2.2. From that model the scaled depth maps and normal maps were created. The following data were prepared:

- Set of RGB and TIR images
- Parameters of the interior orientation of the RGB camera and the TIR camera
- Relative translation and rotation between the RGB camera and the TIR camera
- Referenced point cloud acquired by laser scanner
- Exterior orientation of RGB images
- Depth maps of RGB images
- Normal maps of RGB images

**Figure 11:**
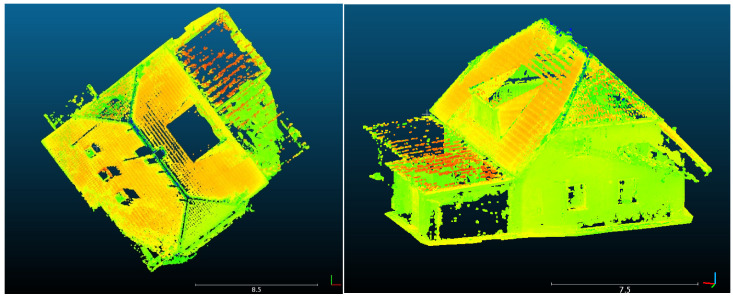
The resulting augmented point cloud visualized in Cloud Compare.

To reduce the processing time of the reprojection method, the point cloud was subsampled to 0.02 m distance between the points, so the point cloud had 1.2 million points in the end. Moreover, only half of the images (94 RGB images) were chosen from the RGB image dataset. According to the previous experiment, where the tolerances were discussed, the tolerance for the depth test was set at 0.025 m (RMSE_xyz_ on control points in photogrammetric model multiplied by five). The tolerance for the normal test was set at 25°. The tolerances could have been set low, because the reprojection method was carried out using a substantial number of images. Then the reprojection process was started. The processing took 12.3 h. The processing time was too high even for a significantly subsampled point cloud. The final resulting point cloud augmented by the temperature information had 511,622 points, which was 42% of the inputted point cloud (see Figure 11). The points were removed either because of low tolerances, or because of imperfections of the 3D model which was used for depth and normal map rendering. Some parts of the model (where the object did not have any texture) were visibly wrong mainly because of a lack of point cloud points from the photogrammetric processing.

**Figure 12:**
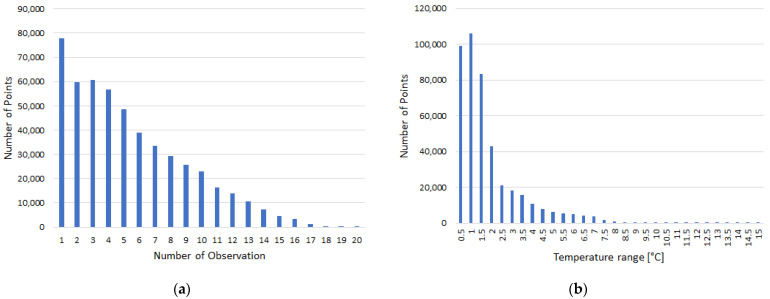
(**a**) Histogram of number of observation values on points of the point cloud. (**b**) Histogram of temperature range values on points of the point cloud.

The augmented point cloud was analyzed. From the total 511,622 augmented points, most of the points (15%) were visible in just one TIR image. According to Figure 12a, 85% of points were visible in more than one image (of a set of 94 images), so, in these cases, the temperature information was determined as a mean of the multiple values. In the case of multiple values, the ranges between the minimum temperature value and the maximum temperature value for each point were analysed. According to Figure 12b, 92% of the observed ranges were under 4 °C, which corresponds to the temperature measurement accuracy of the TIR camera used (±2 °C) [37]. The remaining points can be considered as points with potentially incorrectly assigned temperature, but the high range could be caused by just one outlier.

## 4. Conclusions

The aim of this article was to present and describe the proposed sharpening and reprojection methods in detail. Both implemented methods were also tested by experiment (see Section 3.3). Each method has its problematic questions which have been raised. Experiments were carried to try to answer these questions.

The sharpening method is easy to implement, fast, and suitable mainly for augmented orthophoto and texture of 3D model generation. In the case of the sharpening method, the question of which image band combination is most suitable to use has been raised. According to the experiment described in Section 3.1, the best combination appears to be the RGT combination. This combination obtained slightly fewer tie points compared to the processing of RGB images. When the color information of the result is not needed and when the difference between the minimum and maximum temperature in the dataset is not too large, the RGT combination appears to be a good option. However, the RGT 8-bit combination may not be most suitable for many applications. In the case of a larger difference between the minimum and maximum temperature in the dataset, the 8-bit values may not be enough and a 16-bit RGBT combination should be used. This combination also obtained a respectable number of tie points compared to the RGB images. The RGBT combination also has an advantage in that the full-color information is stored in the resulting augmented orthophoto.

The reprojection method was designed for augmented point cloud generation. Compared to the sharpening method, the processing time is (so far) too high. Among the advantages is that the method can also process laser scanning data (the sharpening method relies on a point cloud generated by photogrammetry). In the reprojection method, the test of visibility is an innovative element. The test of visibility is the key process in the reprojection method. Without the proper visibility test, the final augmentation of the point cloud would be incorrect or misleading. There are many questions regarding the visibility test. The main question is how to set the tolerances in the depth test and normal test. An experiment on the visibility test was carried out and is presented in Section 3.2. The experiment did not provide general rules on how to set the tolerances but gave ideas on how the tolerances can be determined. The tolerances depend on the concrete case. Sometimes it is necessary to set low tolerances, to lose a significant number of points, and to have the certainty that every point will be augmented by correct temperature information. Sometimes it can be more important to preserve every point of the point cloud even when the augmented temperature information may be wrong. In this case, it is possible to use the advantage of the reprojection method, that every point can be augmented by multiple values, and then the incorrect values can be determined and eliminated by outlier detection.

This article sought to present the methods and to raise the main questions regarding the methods. In future research, more extensive testing of the methods on selected objects with different characteristics will be performed.

## Figures and Tables

**Table 1:** Parameters of TIR camera and RGB camera of FLIR E95 [28].

Parameter	TIR Camera	RGB Camera
Focal length (mm)	10	3.29
Sensor size (mm)	7.89 × 5.92	3.67 × 2.74
Resolution (pix)	464 × 348 pix	2592 × 1944
Size of a pixel (mm)	0.017 mm	0.0014 mm
FOV (°)	42° × 32°	53° × 41°

**Table 2:** Resulting parameters of the interior orientation of TIR camera and RGB camera of FLIR E95, determined on a three-level calibration field [28].

		c [pix]	Px [pix]	Py [pix]
TIR camera	3 level cal. field	593.5	−3.3	1.4
RGB camera	3 level cal. field	2481.4	−23.4	27.1

**Table 3:** Resulting parameters of relative translation (ΔX, ΔY, ΔZ) and relative rotation (Δω, Δφ, and Δκ) [28].

	ΔX [m]	ΔY [m]	ΔZ [m]	Δω [°]	Δφ [°]	Δκ [°]
3 level cal. field	−0.0002	−0.0248	−0.0065	−0.833	−0.061	−0.007

**Table 4:** The number of tie points and points of the point cloud from processing of samples of different band combination in comparison to the numbers of processing of RGB images.

		RGT	RTG	TGB	RGBT	TIR
Sample 1 (25 images)	TP	96%	65%	82%	77%	7%
	PC	98%	81%	93%	88%	4%
Sample 2 (10 images)	TP	100%	89%	76%	99%	9%
	PC	98%	84%	99%	81%	6%
Sample 3 (18 images)	TP	95%	75%	84%	84%	12%
	PC	97%	92%	84%	89%	3%

**Table 5:** Testing of tolerances for the depth test and the normal test.

		Reality	Depth Test (Tol = 0.005)	Depth Test (Tol = 0.01)	Depth Test (Tol = 0.015)	Normal Test (Tol = 25°)	Normal Test (Tol = 40°)	Normal Test (Tol = 60°)	Depth Test (Tol = 0.01) and Normal Test (Tol = 40°)
Left Image 13,233	W	Visible	100%	100%	100%	98%	99%	100%	99%
	LJ	Not Visible	1%	3%	4%	3%	10%	27%	1%
	RJ	Visible	84%	96%	99%	43%	68%	87%	66%
Centre image 13,239	W	Visible	100%	100%	100%	95%	99%	100%	99%
	LJ	Visible	17%	33%	49%	22%	48%	76%	16%
	RJ	Visible	6%	13%	21%	25%	50%	78%	7%
Right image 13,217	W	Visible	72%	100%	100%	96%	99%	100%	99%
	LJ	Visible	86%	95%	97%	48%	72%	88%	70%
	RJ	Not Visible	0%	1%	2%	3%	10%	25%	0%
